# Drill Bone with Both Hands: Plunge Depth and Accuracy with 4 Bracing Positions

**DOI:** 10.2106/JBJS.OA.22.00124

**Published:** 2023-03-02

**Authors:** Joseph T. Patterson, Jacob A. Becerra, Andrew Duong, Akhil Reddy, Daniel A. Oakes

**Affiliations:** 1 Department of Orthopaedic Surgery, Keck School of Medicine of the University of Southern California, Los Angeles, California

## Abstract

Bone drilling is a critical skill honed during orthopaedic surgical education. How a bone drill is held and operated (bracing position) may influence drilling performance.

**Methods:**

A prospective study with randomized crossover was conducted to assess the effect of 4 bracing positions on orthopaedic surgical trainee performance in a simulated bone drilling task. Linear mixed effects models considering participant training level, preferred bracing position, height, weight, and drill hole number were used to estimate pairwise and overall comparisons of the effect of each bracing position on 2 primary outcomes of drilling depth and accuracy.

**Results:**

A total of 42 trainees were screened and 19 were randomized and completed the study. Drill plunge depth with a 1-handed drilling position was significantly greater by pairwise comparison to any of the 3 double handed positions tested: a soft tissue protection sleeve in the other hand (0.41 mm, 95% confidence interval [CI] 0.80-0.03, p = 0.031), a 2-handed position with the contralateral small finger on bone and the thumb on the drill (0.42 mm, 95% CI 0.06-0.79, p = 0.018), and a 2-handed position with the contralateral elbow braced against the table (0.40 mm, 95% CI 0.02-0.78, p = 0.038). No position afforded a significant accuracy advantage (p = 0.227). Interactions of participant height with plunge depth and accuracy as well between drill hole number and plunge depth were observed.

**Conclusion:**

Orthopaedic surgical educators should discourage trainees from operating a bone drill using only 1 hand to reduce the risk of iatrogenic injury due to drill plunging.

**Level of Evidence:**

Therapeutic Level II.

## Introduction

Bone drilling is an integral part of orthopedic surgery. Bone drills are frequently used by orthopaedic surgeons to create holes for implant insertion, fracture reduction maneuvers, suture passage, and infection management^1^. Accurate bone drilling is critical to safe and effective surgery^2^. Conversely, “plunging” of a spinning drill bit beyond the intended limit of a drill path can cause serious injury including hemorrhage, compartment syndrome, tendon transection, loss of neurologic function, disability, and reoperation^3-11^.

Bone drilling as a skill is formally incorporated into postgraduate orthopaedic surgical education. The Accreditation Council for Graduate Medical Education considers bone drilling a “critical step” of core orthopaedic procedures and a Level 3 competency milestone required for graduation from an orthopaedic surgery residency^12^. Contemporary orthopaedic surgical education prioritizes instruction in bone drilling in a laboratory workshop environment using synthetic bone models or cadaveric tissue before actual performance on living patients^13-20^. Bone drilling accuracy and depth of plunge while drilling are associated with years of experience and both improve as trainees advance through residency^16,19,21-23^. Equipment factors^1,21-24^ and surgical technique^13-15,17,20,21^ also influence performance.

Limited information is available to guide orthopaedic educators on how to instruct orthopaedic trainees to hold and operate a bone drill to optimize their performance, either in the skills laboratory or in the operating room^13-15^. In addition, the impact of a single simulated bone drilling motor skills workshop on trainee acquisition of this skill is not well described. Establishing the most effective upper-extremity “bracing position(s)” to hold and operate a bone drill may improve trainee performance and present an opportunity to standardize orthopaedic education with positive potential impacts on patient safety and surgical outcomes. We investigated the associations of 4 bracing positions on orthopaedic trainee performance with regard to plunge depth and accuracy in a simulated bone drilling task. We hypothesized that 1 bracing position would be significantly associated with both lowest drill plunge depth and greatest accuracy.

## Methods

A randomized crossover study of simulated surgical techniques with 4 intervention arms was performed at 1 academic teaching institution. The study did not meet criteria for ClinicalTrials.gov registration per Title 42 Code of Federal Regulations (CFR) 11.10 as the interventions were nonclinical. The study was considered exempt per 45 CFR §46.104(d) by our institutional review board before recruitment began. Eligible participants were orthopaedic surgery residents and medical student research associates affiliated with the orthopaedic surgery department. Participants were recruited by email from a departmental mailing list. All participation was voluntary and informed consent was obtained. The participants, principal investigator, residency program director, and all department faculty were blind to study participation and technical performance including drill plunge depth and accuracy measurements to prevent participation or performance from affecting resident education or program advancement. This study was approved by the Human Subjects Division of the University of Southern California Institutional Review Board # HS-21-00125.

An electronic survey of demographic information regarding postgraduate training level by year, sex, height, weight, hand dominance, and preference for an upper-extremity bracing technique was collected before randomization. Participants who completed the survey were then randomly allocated 1 of 24 potential sequences of performing the test with each of the 4 bracing positions using a website (https://www.random.org/). Each bracing position was considered a study arm with all participants randomly crossing over 3 times to all other study arms. Testing occurred at a dedicated clinical skills laboratory in 1 session. The testing station included safety glasses and a synthetic diaphyseal bone model (Generic Bone CHF 0060; Synbone) affixed with wood clamps to a plywood sheet over a block of nonhardening modeling clay (Plastilina #22-7684; Sargent Art) abutting the far cortex. The near cortical surface of the synthetic bone was marked with 3-mm drill start targets at 10-mm intervals on the near cortex and corresponding 3-mm drill exit targets on the opposing far cortex using a printed template and permanent ink. Participants were able to visualize the far cortex from a lateral perspective orthogonal to the drill path but could not see the far cortical target markings which were obscured by clay. System 7 cordless drills with AO quick coupling chucks (Stryker) and 2.5 × 110-mm quick coupling drill bits (DePuy Synthes; Johnson & Johnson) were provided for drilling.

**Fig. 1:**
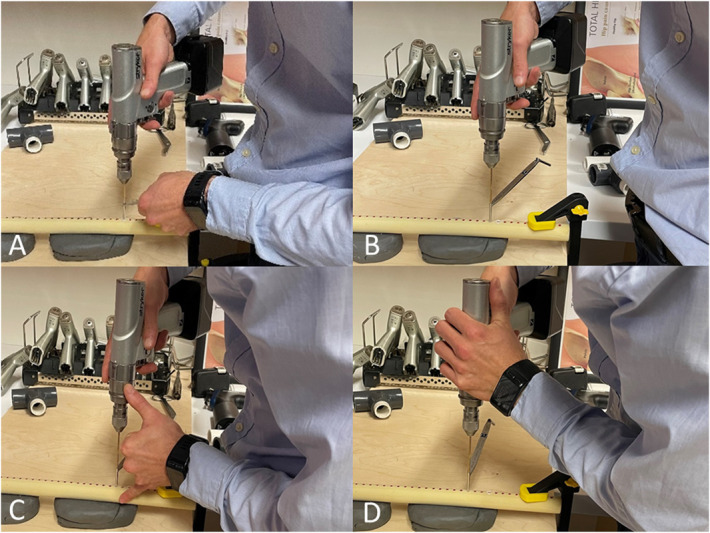
Bracing positions for operating a bone drill: (**Fig. 1-A**) 1-handed drilling position with the contralateral hand holding a soft tissue protection sleeve about the drill bit, (**Fig. 1-B**) 1-handed drilling position without use of contralateral hand for bracing but with the soft tissue protection sleeve in place and free to rotate, (**Fig. 1-C**) 2-handed position with contralateral small finger on bone, and (**Fig. 1-D**) 2-handed position with contralateral elbow bracing on table (**Fig. 1**).

The 4 techniques were defined by ways of holding and operating the cordless bone drill to drill bone. These “bracing positions” included (A) 1-handed drilling position with the contralateral hand holding a soft tissue protection sleeve about the drill bit, (B) 1-handed drilling position without use of contralateral hand for bracing but with the soft tissue protection sleeve in place and free to rotate, (C) 2-handed position with contralateral small finger on bone, and (D) 2-handed position with contralateral elbow bracing on table (Fig. 1). Position (B) was included to reflect the technique of some surgeons who use the soft tissue protection sleeve to attain the designed drill bit trajectory and drill alignment within the hole of a plate for central or eccentric drilling as desired, initiate the drilling to score the bone to prevent drill “walk” or displacement, then switch the nondominant hand to the bone drill before initiating drilling to facilitate greater control of the bone drill while drilling.

Two research associates, second-year medical students, were trained by a fellowship-trained orthopaedic trauma surgeon in the use of the bone drill, instruction of the 4 bracing positions in 2 sessions, and data collection techniques. After demonstration of competence in use and instruction of the techniques, the research associates provided in-person instruction and training in the 4 upper-extremity bracing techniques to all research participants immediately before testing. Participants were shown the bone model including the target markings and instructed to use the bone drill at maximum speed, tuck their elbows adjacent to the torso while drilling, not “bounce” or “peck” the drill bit against either cortex, use a “light hand” to maintain an accurate drill trajectory, listen for pitch change and feel for the loss of resistance as the drill approaches the terminal side of a cortex, drill through the target markings on the far cortex as accurately as possible, and minimize the plunge of the drill bit beyond the far cortex. After instruction, participants were allowed to practice up to 10 minutes before testing.

Participants were then instructed to make 4 bicortical drill holes with each drilling technique, then 4 holes with the next technique, etc., according to their randomly assigned sequence without stopping using their dominant hand to operate the drill with maximal accuracy and minimal plunge of the drill beyond the far cortex into the clay. Randomization was performed to reduce bias attributable to expected motor coordination and skill improvement over the study period. Each participant then exited the skills laboratory. The research associate bisected the clay vertically with a hacksaw (Craftsman 12″ Hacksaw CMHT20138; Stanley Black & Decker), and collected 3 measurements of drill plunge depth into the clay and 3 measurements of drilling accuracy made from the center of drill exit hole to the center of the plantar cortical target in millimeters using an electronic digital caliper (01407A Electronic Digital Caliper; Neiko Tools).

Data were analyzed by a consulting professional statistician as a crossover randomized trial using Tukey pairwise comparisons with the Kenward-Roger method for degrees of freedom and linear mixed effects models (LMEs) considering participant height, training level, pretest preferred hand position, hole number, and a random intercept to account for potential confounding and skill acquisition during the test with model optimization using the Akaike information criterion. Effect modification was explored by introducing interaction terms. We used a significance level of 0.05 in assessing p values. We estimated that 19 participants would be necessary to detect a partial correlation difference of ρ^2^ = 0.4 between bracing position and depth or accuracy measurement using multiple comparisons with the linear mixed effects model including 5 specified control covariates with β = 0.8 and α = 0.025 to considering testing multiple hypothesis with 2 primary outcomes. All statistical analyses were performed with R version 4.0.4 using R packages “effects,” “emmeans,” “lme4,” and “lmerTest.”

## Results

**Fig. 2:**
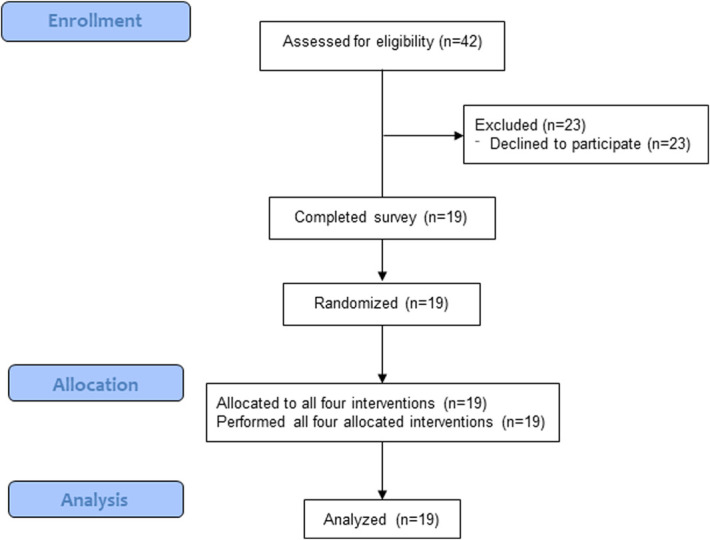
CONSORT flow diagram.

A total of 42 individuals were screened for eligibility. Twenty-three declined to participate and 19 participants who enrolled, randomized, and completed all 4 interventions were included in the analysis (Fig. 2). Participants included 2 medical students, 3 postgraduate year (PGY) 1, 3 PGY2, 3 PGY3, 3 PGY4, and 4 PGY5 orthopaedic surgery residents. Participants were 26.3% female, aged 29.6 ± 2.8 years, height 5′10″ ± 3.8″, weight 170 ± 42.5 lbs, and body mass index 24 ± 3.8 kg/m^2^. 89.5% reported right-hand dominance and 94.7% reported typically using the right hand for drilling. No participant reported a preference for position B before testing.

We revised the LME models of drilling depth and accuracy to address multicollinearity, e.g. correlation between independent variables which might negatively affect the reliability of the statistical inferences made from the models. Removing weight and retaining height (correlated variables) minimized the Akaike information criterion and improved model fit for both models. Analysis of drill plunge depth estimated pairwise point differences of 0.41 mm significantly greater plunge depth (95% CI 0.80-0.03, p = 0.031) with use of position B relative to position A, 0.42 mm (95% CI 0.06-0.79, p = 0.018) relative to position C, and 0.40 mm (95% CI 0.02-0.78, p = 0.038) greater relative to positive D, respectively. There were no significant differences in pairwise plunge depth comparisons between positions A, C, and D (Table I). Overall, no significant advantage was noted when comparing all 4 positions simultaneously (p = 0.227).

**TABLE I:** Drill Plunge Depth and Pairwise Comparisons: Linear Mixed Effects Analysis of the Association of Bracing Position With Drill Plunge Depth, Main-Effects Model

Contrast	Estimated Difference	95% CI	Adjusted p value
Position A − Position B	−0.41	−0.80 to −0.03	0.031
Position A − Position C	0.01	−0.35 to 0.38	>0.999
Position A − Position D	−0.02	−0.37 to 0.34	0.999
Position B − Position C	0.42	0.06 to 0.79	0.018
Position B − Position D	0.40	0.02 to 0.78	0.038
Position C − Position D	0.03	−0.39 to 0.34	0.998

CI = confidence interval.

We did not find any associations, pairwise or overall, between drilling accuracy and bracing positioning (p = 0.969, Table II).

**TABLE II:** Drilling Accuracy and Pairwise Comparisons: Linear Mixed Effects Analysis of the Association of Bracing Position With Drilling Accuracy, Main-Effects Model

Contrast	Estimated Difference	95% CI	Adjusted p value
Position A − Position B	0.41	−0.20 to 1.01	0.292
Position A − Position C	0.37	−0.20 to 0.94	0.323
Position A − Position D	0.27	−0.28 to 0.83	0.563
Position B − Position C	−0.04	−0.61 to 0.54	0.998
Position B − Position D	−0.13	−0.73 to 0.46	0.937
Position C − Position D	−0.09	−0.66 to 0.47	0.971

CI = confidence interval.

We investigated for effect modification. A height × bracing position interaction term significantly improved model fit for both drilling plunge depth (p = 0.007) and accuracy (p = 0.033). An interaction term for bracing position × hole number also provided a significantly better model fit for drill plunge depth (p = 0.023). No other interaction terms significantly improved model fit.

## Discussion

We conducted a randomized 4-arm trial with crossover to investigate effects of how orthopaedic surgery trainees hold and operate a bone drill on drill bit plunge depth and drilling accuracy in a skill task of creating bicortical drill holes in a simulated diaphyseal bone. We were unable to reject our null hypothesis; no single position provided better accuracy and less plunge depth. However, pairwise comparisons identified that a free-hand, 1-handed drilling technique without controlling a soft tissue protection sleeve with the other hand was associated with significantly greater plunge depth in pairwise comparisons to each of the 2-handed drilling techniques.

Orthopaedic surgical procedures involve drilling holes into bone to reduce and stabilize fractures, repair or reconstruct ligaments and tendons, and affix prosthetic components to the skeleton. Accurate and precise drilling of bone is critical to correctly positioning implants and avoiding iatrogenic injury to adjacent structures^2^. Surgical trainees must master the complex skill of operating a bone drill by honing a combination of gross and fine motor coordination: pushing hard enough to cut the bone with the drill while maintaining an accurate trajectory, then metering force and controlling momentum just as the drill is about to exit the bone such that the tip of the drill bit does not “plunge” beyond the limit of the intended drill path. Plunging of a spinning drill bit beyond the far cortex of bone may rarely result in consequential injury to adjacent neurovascular structures. Hemorrhage, compartment syndrome, tendon transection, loss of neurologic function, disability, reoperation, and amputation as consequences of a plunging drill bit have been reported^3-11^. The severity of drill “plunging” as a proxy for risk of iatrogenic injury has been quantified by the depth of drill bit penetration beyond the far cortex, with typical mean depths recorded from experienced surgeons of around 6 mm^2,3,5,24^. Drill plunge depth and accuracy are associated with surgeon training level^13,21,23^, nature of the applied force^23^, bone quality^21^, drill bit sharpness^22^, and drill bit design^1^.

Formal education on equipment maintenance, hand positioning, bracing of the elbows, perpendicular drilling, not bouncing or pecking the drill bit, and maintaining a forward drilling motion during bit withdrawal reduces drilling plunge depth among orthopaedic surgical trainees^13^. However, limited evidence is presently available to guide surgical educators on how exactly a trainee should hold the drill. Langeveld et al. observed that a “shooting” position of the dominant hand on the drill with the long finger on the drill trigger and the nondominant index finger on the far side of the bone or limb as a target is also associated with greater accuracy, despite a 1-handed drilling technique^20^. Ding and Marmor, conversely, noted that 2-handed drilling techniques were associated with the least plunge and lowest variation and that “bouncing” the drill bit against the far cortex was associated with worse performance^14^. The use of large 4.5-mm bits and small sample size may limit the generalizability to typical practice for most osteosynthesis applications. We observed similar depth of plunge and variation as Kazum et al. noted among their orthopaedic trainees using similar equipment including 2.5-mm bits with varied bracing positions^17^.

To the best of our knowledge, no previous study of bone drilling technique has incorporated a randomized design with crossover to address bias resulting from skill improvement during observed testing. We identified an interaction between bracing position and drill hole number which suggests that trainee motor skills as measured by depth and accuracy improve over the course of making just 16 drill holes in a synthetic bone. A fixed sequence of bracing positions with skill acquisition would artifactually make the last bracing position appear most favorable. By randomizing the sequence of bracing positions, we have mitigated bias attributable to this skill learning and to drill bit wear over the test. Our analysis also accounted for height, colinear with weight, which may influence drill inertia and thereby participant control over plunge depth and possibly accuracy with vertical drilling.

There are limitations to this study. Our sample of orthopaedic trainees is nonrandom, small, and does not include experienced surgeons. However, we intentionally recruited a sample with a relatively even distribution of training levels as a training level serves as a presumptive proxy for drilling skill. This sample strategy should improve statistical power and generalizability to orthopaedic surgical education because most residency programs have similar numbers of trainees at each level. All subjects were aware that they were being observed, potentially introducing a Hawthorne effect on performance. The research associates performing the outcome assessments were not blind to bracing sequence or participant characteristics, although investigators and faculty remained blind during and after study completion. As in previous investigations^14,17^, the testing was conducted at a fixed height using a vertical drilling trajectory. This testing setup may negatively affect the performance of short and tall trainees and does not reflect all bone drilling situations encountered in clinical practice. Irrigation of the bone drill, which cools the bone and reduces the risk of thermal necrosis^1^, was not incorporated into this simulation due to concerns about potential water damage to the testing environment. Irrigation may affect drilling performance and the absence of irrigation may limit transportability to bone drilling in the operating room. Participants were permitted a set interval of practice time before observation. Utilization of this opportunity varied, but this variation was not measured and may have influenced the relationships of drill hole number and timing with depth and accuracy. Cutting of the clay block with a hacksaw to measure plunge depth may alter the cavity dimensions and bias the accuracy of the measurements. However, this measurement bias is expected to by uniform across all measurements and participants, with systematic bias attributable to the measurement method affecting accuracy but neither precision nor the significance of the observed differences in plunge depth. Visualizing the depth of the drill bit penetration in clay and using a precise caliper afforded greater precision than use of a depth gauge to measure hole depth in the clay. A depth gauge was observed to deform the clay and generate imprecise measurements.

We conclude that orthopaedic trainees demonstrate greater drill bit plunge depth in a simulated bone drilling task when using a 1-handed bracing position without controlling a soft tissue protection sleeve as compared to 3 other bracing positions. Orthopaedic surgical educators should discourage trainees from operating a bone drill using only 1 hand to reduce the risk of iatrogenic injury due to drill plunging.

### Source of Funding

This work was supported by a Young Investigator Research Development Award from AO Trauma North America as well as grants UL1TR001855 and UL1TR000130 from the National Center for Advancing Translational Science (NCATS) of the U.S. National Institutes of Health. The content is solely the responsibility of the authors and does not necessarily represent the official views of the National Institutes of Health.
